# A Paradoxical Correlation of Cancer-Associated Fibroblasts With Survival Outcomes in B-Cell Lymphomas and Carcinomas

**DOI:** 10.3389/fcell.2018.00098

**Published:** 2018-08-28

**Authors:** Marcela Haro, Sandra Orsulic

**Affiliations:** ^1^Women’s Cancer Program at the Samuel Oschin Comprehensive Cancer Institute, Cedars-Sinai Medical Center, Los Angeles, CA, United States; ^2^Department of Biomedical Sciences, Cedars-Sinai Medical Center, Los Angeles, CA, United States; ^3^Department of Obstetrics and Gynecology, David Geffen School of Medicine, University of California, Los Angeles, Los Angeles, CA, United States

**Keywords:** B cells, B-cell lymphoma, CAFs, cancer-associated fibroblasts, DLBCL, gene signature, ovarian cancer, tumor microenvironment

## Abstract

The tumor microenvironment is increasingly recognized as an active participant in tumor progression. A recent pan-cancer genomic profile analysis has revealed that gene signatures representing components of the tumor microenvironment are robust predictors of survival. A stromal gene signature representing fibroblasts and extracellular matrix components has been associated with good survival in diffuse large B-cell lymphoma (DLBCL). Paradoxically, a closely related gene signature has been shown to correlate with poor survival in carcinomas, including breast, ovarian, pancreatic, and colorectal cancer. To date, there has been no explanation for this paradoxical inverse correlation with survival outcomes in DLBCL and carcinomas. Using public gene data sets, we confirm that the DLBCL stromal gene signature is associated with good survival in DLBCL and several other B-cell lymphomas while it is associated with poor survival in ovarian cancer and several other solid tumors. We show that the DLBCL stromal gene signature is enriched in lymphoid fibroblasts in normal lymph nodes and in cancer-associated fibroblasts (CAFs) in ovarian cancer. Based on these findings, we propose several possible mechanisms by which CAFs may contribute to opposite survival outcomes in B-cell lymphomas and carcinomas.

## Introduction

During the past decade, gene expression profile analyses of frozen tumor pieces have been widely used to quantify various biological characteristics of malignant tumor cells and the microenvironment in which they reside. Individual biological characteristics and dominant molecular pathways in tumors are frequently associated with expression of a defined set of genes, known as a gene expression signature. Since phenotypic features represented by gene expression signatures are sometimes associated with clinical features, such as the length of survival of cancer patients or their response to therapy, gene expression signatures can be used as quantitative predictors of clinical outcomes. A recent pan-cancer PREdiction of Clinical Outcomes from Genomic Profiles (PRECOG) analysis revealed that genes in the tumor microenvironment are better predictors of survival than genes expressed in malignant tumor cells (Gentles et al., 2015). The two most prominent components in the microenvironment of solid tumors are fibroblasts and immune cells (Aran et al., 2017). Generally, in carcinomas, genes expressed in fibroblasts are associated with poor survival while genes expressed in immune cells, particularly leukocytes, are associated with good survival (Gentles et al., 2015). Tumor infiltrating lymphocytes (TILs) and tertiary lymphoid structures (TLS) are generally associated with improved clinical outcomes as evidenced by the improved overall survival and disease-free survival in various types of tumors (Fridman et al., 2012; Dieu-Nosjean et al., 2014; Barnes and Amir, 2017). However, depending on the type of tumor, tumor stage, and location of TILs within the tumor (tumor bed, invasive margin and stroma), different types of TILs have been associated with both positive and negative prognosis. For example, cytotoxic CD8+ T cells, memory T cells, and CD4+ T helper cells are generally associated with a better prognosis, whereas T regulatory cells, tumor associated macrophages, and myeloid-derived suppressor cells are associated with poor prognosis and can promote tumor progression (Fridman et al., 2012; Kitamura et al., 2015; Barnes and Amir, 2017). Furthermore, fibroblasts in the tumor microenvironment are phenotypically heterogeneous and may exhibit both a pro- and anti-tumorigenic phenotype (Augsten, 2014). Thus, the tumor microenvironment is a complex network of interaction between tumor cells and components of the stroma, including the extracellular matrix (ECM), and it is currently unclear which factors in the tumor microenvironment control the quantity and distribution of different immune cell subtypes. Specifically, it is unknown if fibroblasts and immune cells affect prognosis independently or through an interdependent interaction.

The functional interaction between fibroblasts and immune cells has been most thoroughly studied in normal lymph nodes and the spleen, where specialized fibroblasts produce ECM to form a network that allows for lymphocyte movement along the matrix in response to chemokine signaling. The presence of lymphoid fibroblasts is necessary for functional attraction, retention, compartmentalization, and survival of immune cells (Koning and Mebius, 2012). Lymphoid fibroblasts are crucial for lymphocyte homeostasis as well as controlling and expanding the lymphocyte pool (Mueller and Germain, 2009). Lymphoid fibroblasts are also key players in mediating functional immune cell interactions in the lymph nodes through direct contact or via secreted molecules (Chang and Turley, 2015). Follicular dendritic cells (FDC) attract B cells to the germinal center (GC) by secreting C-X-C motif chemokine ligand 13 (CXCL13), while marginal reticular cells (MRC) use a network of follicular conduits to deliver antigens to cognate B cells (Chang and Turley, 2015). By secreting C-C motif chemokine ligands 19 and 21 (CCL19 and CCL21), fibroblastic reticular cells (FRC) recruit mature dendritic cells (DC) and naïve B and T cells to promote cell-cell interactions within the T cell zone (Mueller and Germain, 2009; Brown and Turley, 2015; Fletcher et al., 2015). Recent studies have shown that FRC are important for B-cell homeostasis (Cremasco et al., 2014). This function has been previously ascribed to FDC, however, cell-specific depletion experiments demonstrated that only FRC are crucial for B-cell survival. The mechanism by which FRC support B-cell survival is not entirely clear, but it is thought to involve crosstalk with B cells to control the boundaries of primary B-cell follicles (Cyster, 2010; Mionnet et al., 2013; Cremasco et al., 2014).

Similar to lymphoid fibroblasts in normal lymph nodes, cancer-associated fibroblasts (CAFs) are stromal cells that produce ECM, provide scaffolding, and exert regulatory functions through growth factors, cytokines, and chemokines that can promote tumor growth, angiogenesis, invasion, and metastasis (Kalluri and Zeisberg, 2006; Levental et al., 2009; Lu et al., 2012; Spano and Zollo, 2012; Harper and Sainson, 2014). Recent studies provide evidence that CAFs can also directly or indirectly contribute to immune cell fate and survival (Harper and Sainson, 2014; Costa et al., 2018; Mariathasan et al., 2018; Tauriello et al., 2018). It has recently been shown that a gene signature representing activated CAFs is present in most epithelial tumors (Jia et al., 2016) despite the diversity of resident fibroblasts in different organs and the presence of multiple fibroblast populations within a single tumor type (Costa et al., 2018). Activated CAFs in breast cancer, and possibly in other carcinomas, are associated with immunosuppressive populations of T lymphocytes (Costa et al., 2018). It is unclear if activated CAFs in carcinomas are also associated with immunosuppressive populations of B cells due to poorly defined markers for such cells (Sarvaria et al., 2017). Moreover, studies investigating the associations of B cell subsets with tumor progression using defined B-cell markers have produced conflicting results even within the same tumor type (Guy et al., 2016). An insufficient understanding of the roles of B cells in carcinomas has hindered the development of rational clinical trials targeting B-cells in carcinomas. The remarkable success of B-cell depletion with the cluster of differentiation 20 (CD20) monoclonal antibody, rituximab, in lymphomas and rheumatoid arthritis has sparked interest in rituximab and other B-cell targeted antibodies as possible therapies in carcinomas (Gunderson and Coussens, 2013). Although many carcinomas have significant B cell infiltration (Germain et al., 2014), clinical trials have shown limited benefits of B-cell depletion in carcinomas (Barbera-Guillem et al., 2000; Aklilu et al., 2004), possibly because B cells can have pro-tumorigenic or anti-tumorigenic properties depending on their maturation stage and other conditions that have not yet been defined (Sarvaria et al., 2017).

## The DLBCL Stromal-1 Gene Signature is Inversely Correlated with Survival Outcomes in B-Cell Lymphomas and Other Solid Tumors

Using expression profile analysis of DLBCL biopsy samples from treatment-naïve newly diagnosed patients, Lenz et al. identified two stromal gene signatures, stromal-1 and stromal-2, of which the stromal-1 gene signature was found to be associated with good survival in DLBCL patients (Lenz et al., 2008). However, gene signatures similar to the DLBCL stromal-1 gene signatures have been associated with poor survival in carcinomas, including ovarian cancer (Cheon et al., 2014), breast cancer (Farmer et al., 2009), colorectal cancer (Calon et al., 2015; Isella et al., 2015), and pancreatic cancer (Moffitt et al., 2015). To systematically explore the association of the DLBCL stromal-1 gene signature with survival in cancer patients, we used PRECOG, a pan-cancer database of expression signatures in which each tumor type is represented by multiple independent expression profile data sets and associated survival data. This extensive database is ideal for multi-data set validation of prognostic signatures that have been identified in individual data sets. Using the DLBCL stromal-1 gene signature represented by 50 genes (Lenz et al., 2008), we confirmed that the signature is associated with poor survival in carcinomas and brain tumors and good survival in DLBCL and several other B-cell lymphomas (**Table 1**). This pattern of inverse association with survival between B-cell lymphomas and carcinomas/brain tumors was specific to the DLBCL stromal-1 gene signature, and was not associated with the DLBCL stromal-2 gene signature represented by 34 genes (Lenz et al., 2008) (data not shown).

**Table 1:** DLBCL “stromal-1” signature genes are inversely correlated with survival outcomes in B-cell lymphomas and other malignancies.

	B-cell lymphoma						Solid tumor					
Gene	BL	CLL	DLBCL	FL	MCL	MM	Bladder	Astro cytoma	Glioma	Colon	Head and neck	Ovarian
ACTN1	-0.928	-3.216	-6.211	-1.901	-0.94	0.658	3.312	3.22	4.557	2.36	1.988	1.552
ADAM12	0.746	-0.084	-7.809	-1.749	-0.866	-0.395	0.537	1.653	4.405	1.675	2.051	2.99
BGN	0.842	1.309	-4.115	-1.775	0	-2.627	1.438	2.341	3.643	2.33	3.559	3.09
CEBPA	-1.516	-3.127	-5.644	-1.639	0	-0.977	1.001	-0.041	2.652	-2.664	-1.578	-1.442
COL13A1	-0.313	-1.513	-2.402	0.332	0	-0.001	2.23	2.006	1.613	2.164	1.74	0.893
COL16A1	-0.481	0.252	-3.89	-0.6	0.333	-0.477	2.214	2.49	5.005	-0.546	1.263	4.542
COL1A1	0.349	-1.476	-4.621	-1.581	0	-1.951	3.592	3.326	3.77	1.544	3.354	3.929
COL1A2	-0.097	-0.879	-6.264	-1.605	0	-0.573	2.745	4.432	4.391	2.42	2.634	3.771
COL5A1	0.715	-0.675	-3.366	0.127	0	-0.467	1.957	3.528	4.438	2.328	3.686	3.65
COL5A2	0.969	1.124	-3.962	-1.597	0	-0.777	3.47	3.588	7.322	2.437	3.26	5.256
COL6A2	0.677	-1.368	-3.719	-0.749	-1.415	0.14	2.369	4.591	5.693	1.301	3.12	2.11
COL6A3	1.194	-0.129	-4.502	-1.442	1.37	2.684	1.282	3.005	3.071	2.403	3.141	3.178
COL8A2	-0.212	-0.894	-3.046	0.069	0	-0.905	-0.085	2.942	3.077	-0.007	1.779	2.908
CSF2RA	-1.84	0	-2.861	0	0	-2.39	-0.046	0.193	0	0	0	-1.959
CTGF	-0.5	0.796	-5.525	-0.73	-1.387	-0.775	1.651	1.676	-1.132	2.024	2.381	2.974
CYR61	1.159	0.092	-1.865	0.074	1.837	-0.123	3.342	1.159	3.807	1.678	1.757	3.607
DCN	0.819	0.185	-3.731	-0.026	0	-0.794	0.472	1.113	2.414	1.303	0.917	4.604
EFEMP2	1.823	1.113	-2.797	0.307	0	-5.014	2.112	4.044	7.62	1.684	3.53	2.576
EMP2	-0.057	0.044	-4.122	0.147	0	-0.579	-1.125	4.55	2.985	-0.368	0.452	-1.446
FAP	-1.551	0.374	-7.496	-0.76	-1.266	-0.536	3.522	2.321	3.736	2.366	2.874	4.814
FBN1	1.125	1.079	-4.907	-1.854	0	-0.044	2.151	1.518	2.239	2.311	1.906	4.676
FN1	-1.025	-0.496	-5.638	-1.852	-1.352	2.973	3.251	2.852	5.499	2.628	2.46	4.439
GPNMB	-1.638	-0.153	-6.899	0.513	0	1.112	1.281	3.946	5.214	1.74	-2.745	1.476
HSPG2	-0.267	2.244	-2.792	-1.63	0	0.845	-0.02	4.261	2.989	1.313	2.108	2.396
IL1R1	-1.566	-2.791	-4.858	-0.432	0.804	-1.789	-0.186	1.194	1.217	1.275	0.897	-0.137
ITGAV	0.897	-2.698	-6.933	0.614	-2.033	-0.212	0.402	0.945	0.226	2.253	1.503	1.792
ITGB2	-1.522	-2.053	-5.68	0.558	0.343	-1.803	0.886	0.4	4.299	-0.086	-2.064	-2.339
KITLG	0.896	-0.172	-1.923	1.04	-1.197	0.454	1.113	-0.331	1.091	1.164	-0.721	-0.504
LAMA4	0.445	2.207	-3.683	0.453	0	-3.155	2.474	0.028	3.397	2.415	2.021	2.168
LAMB2	-0.635	0.504	-1.974	-1.052	0	-0.728	0.926	1.686	5.906	0.913	1.836	2.326
LAMB3	1.291	-1.315	-2.703	0.256	0	0.265	-0.927	1.977	3.542	1.516	2.039	-1.966
LOXL1	-1.453	-1.007	-4.202	-1.287	0	-1.92	0.711	3.9	6.299	1.697	0.751	3.664
LTBP2	0.219	-1.562	-7.565	-0.187	0	-1.848	2.849	1.197	3.314	0.542	2.718	1.541
LUM	-0.357	-1.043	-5.663	-0.089	0	-1.859	1.442	3.796	3.723	1.447	1.428	4.841
MFAP2	0.862	0.01	-2.835	0.608	0	-0.68	3.151	3.543	3.011	0.874	1.666	5.462
MMP14	-1.105	2.746	-3.319	0.69	0.681	-1.647	2.046	1.787	4.691	1.786	1.168	2.297
MMP2	-1.227	-0.269	-5.709	-1.128	0.014	-0.545	0.66	1.792	3.631	1.567	3.12	3.084
MMP9	-0.819	-1.238	-7.734	-0.401	-0.12	-0.892	1.8	2.739	5.06	-0.723	0.039	-3.208
PDGFC	0.62	-3.08	-4.268	0.632	0	-0.486	2.788	-3.419	3.639	1.987	2.096	-0.167
PLAU	-1.723	-1.701	-7.712	0.205	0.528	-0.749	2.515	2.302	4.592	0.627	1.521	2.334
POSTN	1.565	0.675	-5.031	-1.266	-0.77	-1.157	3.246	2.76	5.46	2.632	2.092	4.696
SDC2	-0.209	-1.963	-3.763	-0.47	-0.383	-0.664	-1.091	1.405	5.736	2.239	1.659	1.424
SERPINH1	-1.173	2.067	-2.912	-1.224	0	1.565	1.422	3.846	5.397	3.044	2.065	2.07
SPARC	0.487	-3.125	-7.236	-1.599	1.012	-2.767	2.24	-1.998	-0.074	2.412	2.933	4.188
TGFB1I1	-0.842	-1.479	-2.367	0.662	0	-1.787	1.518	2.783	4.58	1.523	3.557	4.265
THBS1	1.462	-3.212	-2.038	-1.38	0.238	-1.674	1.673	2.947	3.122	0.799	2.328	3.565
TIMP2	-0.677	-2.448	-1.399	1.006	0.343	0.83	2.608	1.584	1.251	2.73	2.271	2.495
VCAN	1.459	-3.803	-3.177	-0.588	0	-2.078	3.133	-3.546	-3.171	2.264	2.238	4.277

*Analysis of the DLBCL “stromal-1” geneset in the PREdiction of Clinical Outcomes from Genomic Profiles (PRECOG) public dataset (https://precog.stanford.edu). Each gene is assigned z scores associated with survival in different cancer types. Scores less than or equal to zero (red) are associated with good survival while positive scores (blue) are associated with poor survival. BL, Burkitt lymphoma; CLL, chronic lymphocytic leukemia; DLBCL, diffuse large B-cell lymphoma; FL, follicular lymphoma; MCL, mantle cell lymphoma; MM, multiple myeloma.*

## In Normal Lymph Nodes, DLBCL Stromal-1 and Stromal-2 Gene Signatures are Enriched in Stromal Fibroblasts and Endothelial Cells, Respectively

**FIGURE 1:**
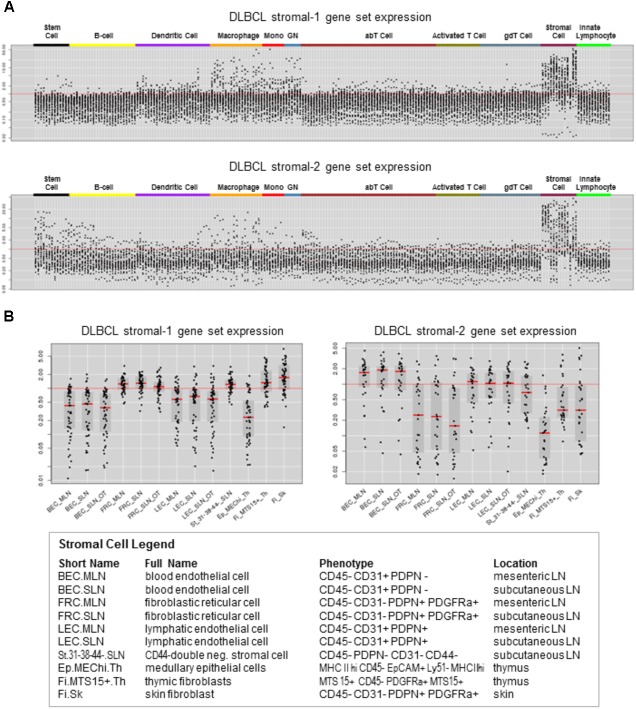
DLBCL stromal-1 and stromal-2 signature genes are enriched in different stromal cell types. Expression of the DLBCL stromal-1 and stromal-2 signature genes in the Immunological Genome Project (ImmGen) data set. **(A)** Gene expression values normalized across 249 mouse immunological cell types. **(B)** Detailed view of gene expression values normalized to the stromal cell types shown in the legend. The graphs were generated using data from ImmGen (http://www.immgen.org).

To identify immune cell types that express the DLBCL stromal-1 and stromal-2 signature genes, we looked for enrichment of these genes in the transcriptomes of 249 normal immunological cell types that had been isolated from mice and characterized by the Immunological Genome Project (ImmGen) (Heng and Painter, 2008; Shay and Kang, 2013). This analysis identified stromal cells as the most likely source of both gene signatures, although some of the genes were also expressed in macrophages, monocytes, granulocytes, and stem cells (**Figure 1A**). Closer examination of the stromal cell subtypes revealed that the DLBCL stromal-1 and stromal-2 signature genes were preferentially expressed in different types of stromal cells. DLBCL stromal-1 signature genes were particularly enriched in cells characterized by expression of podoplanin (PDPN) and platelet-derived growth factor receptor α (PDGFRα), including FRC from mesenteric and subcutaneous lymph nodes and the so-called double-negative stromal cells, while stromal-2 signature genes were enriched in blood and lymphatic endothelial cells (**Figure 1B**).

## The DLBCL Stromal-1 Gene Signature is Enriched in Ovarian CAFs

**FIGURE 2:**
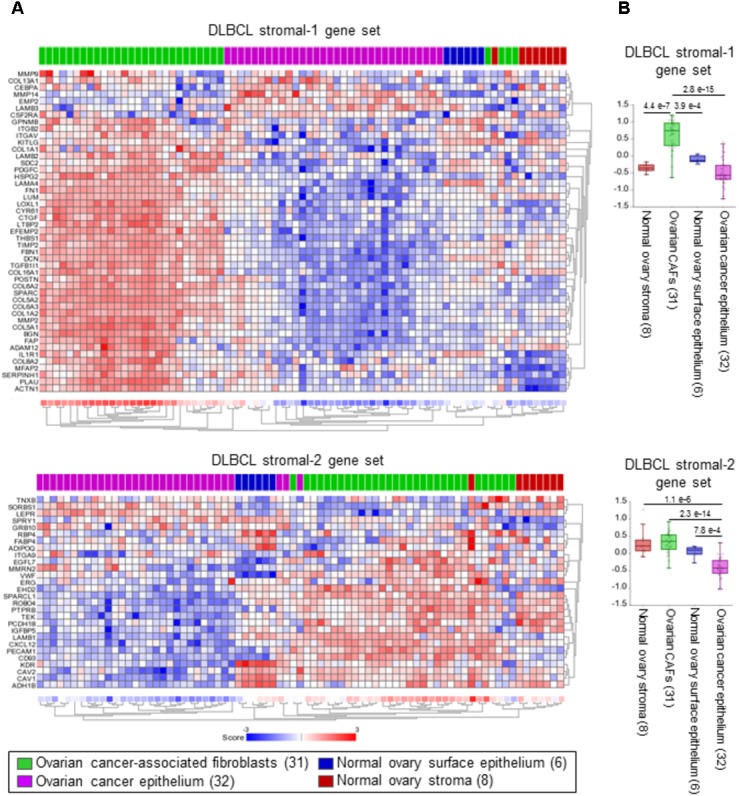
DLBCL stromal-1 signature genes are enriched in cancer-associated fibroblasts (CAFs). **(A)** Non-centered gene set clustering analysis of the stromal and epithelial cell types in ovarian cancer and the normal ovary in the GSE40595 dataset using the DLBCL stromal-1 and stromal-2 gene sets. The number of samples in each group is indicated in parentheses. The gene set clustering analysis and image acquisition was performed using the R2 Genomics Analysis and Visualization Platform (https://hgserver1.amc.nl). **(B)** The same data are shown as box dot plots with *P*-values for differential expression of the DLBCL stromal-1 and stromal-2 gene signatures in different cell types.

To identify cells that express the DLBCL stromal-1 and stromal-2 signature genes in an epithelial tumor, we selected ovarian cancer because of the existing microarray data set (GSE40595) in which a large number of ovarian cancers have been laser capture microdissected into epithelial and stromal components (Yeung et al., 2013). For comparison with normal tissue, a small number of samples in this data set were microdissected from the normal ovary epithelium and stroma (Yeung et al., 2013). Our gene signature enrichment analysis revealed strong enrichment of the DLBCL stromal-1 gene signature in CAFs in comparison to cancer cells, normal ovary fibroblasts, and normal ovary epithelial cells (**Figure 2**). The DLBCL stromal-2 gene signature was enriched in CAFs but also in the normal ovary stroma (**Figure 2**).

## Possible Mechanisms by Which CAFs Contribute to Inverse Survival Outcomes in B-Cell Lymphomas and Carcinomas

It is unusual for a gene signature to be associated with inverse survival outcomes in B-cell lymphomas and carcinomas. This is unlikely to be a technical error related to microarray technology as several individual genes from the DLBCL stromal-1 signature have been validated as predictors of good survival in DLBCL by independent technologies, such as immunohistochemistry and qPCR in formalin-fixed paraffin-embedded tissues (Lossos et al., 2004; Meyer et al., 2011; Tekin et al., 2016). Similarly, various technologies have been used to validate many of the signature genes as predictors of poor survival in carcinomas (Farmer et al., 2009; Cheon et al., 2014; Calon et al., 2015; Isella et al., 2015; Moffitt et al., 2015; Jia et al., 2016). While the mechanism by which the DLBCL stromal-1 signature genes could contribute to good survival in DLBCL is still unclear, multiple mechanisms by which CAFs contribute to poor outcomes in carcinomas have been proposed, including the promotion of tumor growth, angiogenesis, invasion and metastasis, the provision of protective niches for cancer stem cells, and the obstruction of access of chemotherapies and immunotherapies (Jain, 2013; Kalluri, 2016). Here, we will specifically focus on the possible direct or indirect roles of CAFs that could contribute to inverse survival outcomes in DLBCL and carcinomas.

Cancer-associated fibroblasts share structural and molecular features with the reticular fiber networks of secondary lymphoid organs, which are known to guide and compartmentalize specific immune cell types and play key roles in mediating functional immune cell interactions (Acton et al., 2012; Astarita et al., 2012; Cremasco et al., 2014; Chang and Turley, 2015; Fletcher et al., 2015; Turley et al., 2015). However, in addition to being sites in which immune responses are initiated, secondary lymphoid organs are also sites that foster immune privilege that prevents autoimmunity by inducing tolerance and deleting autoreactive T cells, suppressing effector T cell proliferation, and supporting regulatory T cells (Fletcher et al., 2011, 2014, 2015; Brown and Turley, 2015). Currently, lymph node fibroblasts are being explored for their therapeutic potential to circumvent unwanted inflammation in autoimmune diseases, sepsis, and graft rejection after organ transplantation (Fletcher et al., 2011, 2014, 2015). Based on the molecular similarity between CAFs and lymph node fibroblasts, we propose that CAFs primarily play an immunosuppressive role in tumors using similar molecular mechanisms to those used by lymph node fibroblasts in regulating immune cell tolerance and homeostasis. In support of this hypothesis, CAF-derived factors have been shown to contribute to immune editing *in vivo* to avoid tumor detection and rejection by the host immune system (Stover et al., 2007; Kraman et al., 2010). Specific to B cells, several *in vitro* models have shown the ability of different types of fibroblasts to modulate B cell differentiation, activation, and function. Adipose tissue-derived fibroblasts have been shown to suppress plasmablast formation and induce formation of regulatory B cells (Franquesa et al., 2015) while rheumatoid synovial fibroblasts have been shown to induce immunoglobulin (Ig) class-switch recombination and IgG/IgA production in IgD+ B cells (Bombardieri et al., 2011). We envision that the immunoregulatory functions of CAFs may lead to improved survival in DLBCL and other B-cell lymphomas where malignant cells themselves are subject to functional alteration. In contrast, immunosuppression by CAFs in carcinomas may lead to an ineffective immune defense against malignant cells, which is associated with poor survival.

**FIGURE 3:**
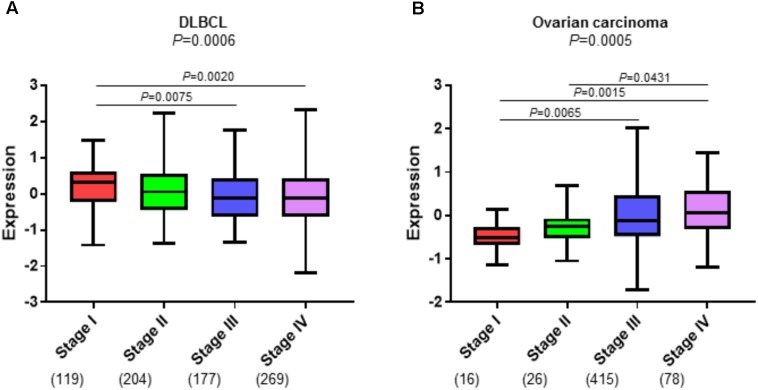
CAFs have an inverse association with tumor stage in DLBCL and ovarian carcinoma. Enrichment of the DLBCL stromal-1 gene signature in progression stages I-IV in **(A)** three DLBCL microarray datasets (GSE10846, GSE87371, and GSE4475) that were combined into one dataset, and **(B)** The Cancer Genome Atlas (TCGA) ovarian carcinoma dataset (https://cancergenome.nih.gov). The gene signature enrichment analysis was performed using the R2 Genomics Analysis and Visualization Platform (https://hgserver1.amc.nl). The y axis shows relative enrichment of the DLBCL stomal-1 gene signature. The x axis shows tumor stage. The number of samples for each tumor stage is indicated in parentheses.

Cancer-associated fibroblasts are also capable of modifying the immune landscape by selective attraction, recruitment, retention, activation, and suppression of different immune cell types (Karin, 2010; Raz and Erez, 2013; Harper and Sainson, 2014). Recent studies provide evidence that CAFs can directly contribute to immune cell fate and survival (Harper and Sainson, 2014). In mouse models, CAFs have been shown to attract macrophages, neutrophils, and subsets of T cells that promote tumor progression (Silzle et al., 2003; Grum-Schwensen et al., 2010; Elkabets et al., 2011). One possible underlying mechanism for the association of the DLBCL stromal-1 gene signature with good survival in patients with DLBCL is that fibroblasts and the associated ECM attract and trap malignant B cells thereby impeding their spread to new anatomical locations. We show a small but consistent inverse association of the DLBCL stromal-1 gene signature expression with DLBCL tumor stage (a measure of lymph node groups and extranodal sites to which malignant cells have metastasized) (**Figure 3A**). The decrease in stromal gene signature expression in the later stages of DLBCL may indicate that the stroma plays a role in localizing the lymphoma cells to the lymph nodes during the earlier stages of the disease. In contrast, DLBCL stromal-1 gene signature expression is typically increased with increased tumor stage in epithelial carcinomas, such as ovarian cancer (**Figure 3B**). The increase in CAFs in the later stages of carcinomas may prevent immune cells from reaching the tumor parenchyma by trapping the immune cells in the stroma thereby preventing an anti-tumor response. A recent study of immune cell infiltration in metastatic urothelial carcinomas showed that patients whose tumors were classified as immune-excluded (immune cells localized in the CAF-rich stroma) had increased disease progression and decreased response to immunotherapy (Mariathasan et al., 2018). Therefore, we hypothesize that CAFs aid in retaining DLBCL in the lymph node, which is associated with better prognosis, whereas in carcinomas CAFs trap immune cells, which is associated with decreased anti-tumor immune activity and a worse prognosis.

One of the key modulators of the cancer microenvironment is the multifunctional cytokine, transforming growth factor β (TGFβ). TGFβ induces CAF activation and fibroblast-to-myofibroblast transition with consequent linearization of collagen fibers and stiffening of the ECM. In turn, activated CAFs induce TGFβ signaling to perpetually maintain the activated state (Calon et al., 2014; Beach et al., 2016; Erdogan and Webb, 2017). Consistent with the DLBCL stromal-1 signature representing CAFs, our Ingenuity Pathway Analysis (IPA) of the DLBCL gene signatures implicates TGFβ signaling as the main upstream regulator of the DLBCL stromal-1 gene signature (**Table 2**). In carcinomas, TGFβ has been shown to promote tumor progression by inhibiting immunosurveillance through multiple mechanisms (Flavell et al., 2010; Sheng et al., 2015), including the recruitment of macrophages (Byrne et al., 2008) and limited efficacy of immunotherapy by excluding CD8^+^ T cells from the tumor parenchyma (Mariathasan et al., 2018; Tauriello et al., 2018). It is likely that TGFβ also plays an immunosuppressive role in lymphomas. However, TGFβ is also a potent negative regulator of B-cell survival, proliferation, activation, and differentiation (Sanjabi et al., 2017). Stroma-derived TGFβ has been shown to induce senescence and apoptosis in mouse models of B-cell lymphoma (Reimann et al., 2010; Stelling et al., 2018). Thus, the DLBCL stromal-1 gene signature may be primarily associated with tumor-promoting immunosuppression in carcinomas, while the same immunosuppression may lead to the eradication of B cells, which represent the malignant component of B-cell lymphoma.

**Table 2:** Upstream regulators of genes in the DLBCL stromal gene signature-1 and stromal gene signature-2.

Upstream regulator	Molecule type	*p*-value of overlap
**DLBCL stromal-1 gene signature**	
TGFB1	Growth factor	4.78E-31
COLQ	Other	2.70E-20
Bleomycin	Chemical drug	1.97E-18
SPDEF	Transcription regulator	2.73E-18
Tgf beta	Group	3.95E-18
TGFB3	Growth factor	8.04E-18
TNF	Cytokine	1.53E-17
**DLBCL stromal-2 gene signature**	
KLF2	Transcription regulator	1.89E-09
Rosiglitazone	Chemical drug	5.82E-09
VEGFA	Growth factor	5.90E-09
PPARG	Ligand-dependent nuclear receptor	1.36E-08
10E, 12Z-octadecadienoic acid	Chemical – endogenous Mammalian	4.98E-08
WNT3A	Cytokine	6.02E-08
MGEA5	Enzyme	1.08E-07

*The identification of upstream regulators was done using Ingenuity Pathway Analysis (www.qiagenbioinformatics.com/products/ingenuity-pathway-analysis/).*

## Conclusion

Past clinical trials have taught us that successful targeted therapies in one disease do not always yield the desired results in another disease despite the presence of the same target. One example is the poor response of B-cell-infiltrated carcinomas to rituximab, which has shown remarkable success in lymphomas and rheumatoid arthritis. The opposite survival outcomes associated with the presence of stromal cells in B-cell lymphomas and carcinomas should serve as a warning that targeting the tumor microenvironment may produce opposite effects in B-cell lymphomas and carcinomas.

## Database Links

GEO Data Sets (https://www.ncbi.nlm.nih.gov/gds) Immunological Genome Project (https://www.immgen.org) PRECOG – PREdiction of Clinical Outcomes from Genomic Profiles (https://precog.stanford.edu) R2: Genomics Analysis and Visualization Platform (http://hgserver1.amc.nl) The Cancer Genome Atlas Project (https://cancergenome.nih.gov).

## Author Contributions

SO analyzed the public data sets. SO and MH wrote the manuscript.

## Conflict of Interest Statement

The authors declare that the research was conducted in the absence of any commercial or financial relationships that could be construed as a potential conflict of interest.

## Abbreviations

CAF: cancer-associated fibroblast
CD: cluster of differentiation
CXCL: C-X-C motif chemokine ligand
DC: dendritic cells
DLBCL: diffuse large B-cell lymphoma
ECM: extracellular matrix
FDC: follicular dendritic cells
FRC: fibroblastic reticular cells
GC: germinal center
Ig: immunoglobulin
ImmGen: immunological genome project
IPA: ingenuity pathway analysis
MRC: marginal reticular cells
NK: natural killer
PDGFRα: platelet-derived growth factor receptor α
PDPN: podoplanin
PRECOG: PREdiction of clinical outcomes from genomic profiles
TCGA: the Cancer genome atlas project
TGFβ: transforming growth factor β
TIL: tumor infiltrating lymphocyte
TLS: tertiary lymphoid structure

## References

[B1] Acton S. E. Astarita J. L. Malhotra D. Lukacs-Kornek V. Franz B. Hess P. R. (2012). Podoplanin-rich stromal networks induce dendritic cell motility via activation of the C-type lectin receptor CLEC-2. *Immunity* 37 276–289. 10.1016/j.immuni.2012.05.022 22884313 PMC3556784

[B2] Aklilu M. Stadler W. M. Markiewicz M. Vogelzang N. J. Mahowald M. Johnson M. (2004). Depletion of normal B cells with rituximab as an adjunct to IL-2 therapy for renal cell carcinoma and melanoma. *Ann. Oncol.* 15 1109–1114. 10.1093/annonc/mdh280 15205206

[B3] Aran D. Hu Z. Butte A. J. (2017). xCell: digitally portraying the tissue cellular heterogeneity landscape. *Genome Biol.* 18:220. 10.1186/s13059-017-1349-1 29141660 PMC5688663

[B4] Astarita J. L. Acton S. E. Turley S. J. (2012). Podoplanin: emerging functions in development, the immune system, and cancer. *Front. Immunol.* 3:283. 10.3389/fimmu.2012.00283 22988448 PMC3439854

[B5] Augsten M. (2014). Cancer-associated fibroblasts as another polarized cell type of the tumor microenvironment. *Front. Oncol.* 4:62. 10.3389/fonc.2014.00062 24734219 PMC3973916

[B6] Barbera-Guillem E. Nelson M. B. Barr B. Nyhus J. K. May K. F. Jr. Feng L. (2000). B lymphocyte pathology in human colorectal cancer. Experimental and clinical therapeutic effects of partial B cell depletion. *Cancer Immunol. Immunother.* 48 541–549. 10.1007/PL00006672 10630306 PMC11037163

[B7] Barnes T. A. Amir E. (2017). HYPE or HOPE: the prognostic value of infiltrating immune cells in cancer. *Br. J. Cancer* 117 451–460. 10.1038/bjc.2017.220 28704840 PMC5558691

[B8] Beach J. A. Aspuria P. J. Cheon D. J. Lawrenson K. Agadjanian H. Walsh C. S. (2016). Sphingosine kinase 1 is required for TGF-beta mediated fibroblastto- myofibroblast differentiation in ovarian cancer. *Oncotarget* 7 4167–4182. 10.18632/oncotarget.6703 26716409 PMC4826197

[B9] Bombardieri M. Kam N. W. Brentano F. Choi K. Filer A. Kyburz D. (2011). A BAFF/APRIL-dependent TLR3-stimulated pathway enhances the capacity of rheumatoid synovial fibroblasts to induce AID expression and Ig class-switching in B cells. *Ann. Rheum. Dis.* 70 1857–1865. 10.1136/ard.2011.150219 21798884

[B10] Brown F. D. Turley S. J. (2015). Fibroblastic reticular cells: organization and regulation of the T lymphocyte life cycle. *J. Immunol.* 194 1389–1394. 10.4049/jimmunol.1402520 25663676 PMC4324549

[B11] Byrne S. N. Knox M. C. Halliday G. M. (2008). TGFbeta is responsible for skin tumour infiltration by macrophages enabling the tumours to escape immune destruction. *Immunol. Cell Biol.* 86 92–97. 10.1038/sj.icb.7100116 17768418

[B12] Calon A. Lonardo E. Berenguer-Llergo A. Espinet E. Hernando-Momblona X. Iglesias M. (2015). Stromal gene expression defines poor-prognosis subtypes in colorectal cancer. *Nat. Genet.* 47 320–329. 10.1038/ng.3225 25706628

[B13] Calon A. Tauriello D. V. Batlle E. (2014). TGF-beta in CAF-mediated tumor growth and metastasis. *Semin. Cancer Biol.* 25 15–22. 10.1016/j.semcancer.2013.12.008 24412104

[B14] Chang J. E. Turley S. J. (2015). Stromal infrastructure of the lymph node and coordination of immunity. *Trends Immunol.* 36 30–39. 10.1016/j.it.2014.11.003 25499856

[B15] Cheon D. J. Tong Y. Sim M. S. Dering J. Berel D. Cui X. (2014). A collagen-remodeling gene signature regulated by TGF-beta signaling is associated with metastasis and poor survival in serous ovarian cancer. *Clin. Cancer Res.* 20 711–723. 10.1158/1078-0432.CCR-13-1256 24218511 PMC3946428

[B16] Costa A. Kieffer Y. Scholer-Dahirel A. Pelon F. Bourachot B. Cardon M. (2018). Fibroblast heterogeneity and immunosuppressive environment in human breast cancer. *Cancer Cell* 33 463.e10–479.e10. 10.1016/j.ccell.2018.01.011 29455927

[B17] Cremasco V. Woodruff M. C. Onder L. Cupovic J. Nieves-Bonilla J. M. Schildberg F. A. (2014). B cell homeostasis and follicle confines are governed by fibroblastic reticular cells. *Nat. Immunol.* 15 973–981. 10.1038/ni.2965 25151489 PMC4205585

[B18] Cyster J. G. (2010). B cell follicles and antigen encounters of the third kind. *Nat. Immunol.* 11 989–996. 10.1038/ni.1946 20959804

[B19] Dieu-Nosjean M. C. Goc J. Giraldo N. A. Sautes-Fridman C. Fridman W. H. (2014). Tertiary lymphoid structures in cancer and beyond. *Trends Immunol.* 35 571–580. 10.1016/j.it.2014.09.006 25443495

[B20] Elkabets M. Gifford A. M. Scheel C. Nilsson B. Reinhardt F. Bray M. A. (2011). Human tumors instigate granulin-expressing hematopoietic cells that promote malignancy by activating stromal fibroblasts in mice. *J. Clin. Invest.* 121 784–799. 10.1172/JCI43757 21266779 PMC3026724

[B21] Erdogan B. Webb D. J. (2017). Cancer-associated fibroblasts modulate growth factor signaling and extracellular matrix remodeling to regulate tumor metastasis. *Biochem. Soc. Trans.* 45 229–236. 10.1042/bst20160387 28202677 PMC5371349

[B22] Farmer P. Bonnefoi H. Anderle P. Cameron D. Wirapati P. Becette V. (2009). A stroma-related gene signature predicts resistance to neoadjuvant chemotherapy in breast cancer. *Nat. Med.* 15 68–74. 10.1038/nm.1908 19122658

[B23] Flavell R. A. Sanjabi S. Wrzesinski S. H. Licona-Limon P. (2010). The polarization of immune cells in the tumour environment by TGFbeta. *Nat. Rev. Immunol.* 10 554–567. 10.1038/nri2808 20616810 PMC3885992

[B24] Fletcher A. L. Acton S. E. Knoblich K. (2015). Lymph node fibroblastic reticular cells in health and disease. *Nat. Rev. Immunol.* 15 350–361. 10.1038/nri3846 25998961 PMC5152733

[B25] Fletcher A. L. Elman J. S. Astarita J. Murray R. Saeidi N. D’Rozario J. (2014). Lymph node fibroblastic reticular cell transplants show robust therapeutic efficacy in high-mortality murine sepsis. *Sci. Transl. Med.* 6:249ra109. 10.1126/scitranslmed.3009377 25122637 PMC4415170

[B26] Fletcher A. L. Malhotra D. Turley S. J. (2011). Lymph node stroma broaden the peripheral tolerance paradigm. *Trends Immunol.* 32 12–18. 10.1016/j.it.2010.11.002 21147035 PMC3163075

[B27] Franquesa M. Mensah F. K. Huizinga R. Strini T. Boon L. Lombardo E. (2015). Human adipose tissue-derived mesenchymal stem cells abrogate plasmablast formation and induce regulatory B cells independently of T helper cells. *Stem Cells* 33 880–891. 10.1002/stem.1881 25376628

[B28] Fridman W. H. Pages F. Sautes-Fridman C. Galon J. (2012). The immune contexture in human tumours: impact on clinical outcome. *Nat. Rev. Cancer* 12 298–306. 10.1038/nrc3245 22419253

[B29] Gentles A. J. Newman A. M. Liu C. L. Bratman S. V. Feng W. Kim D. (2015). The prognostic landscape of genes and infiltrating immune cells across human cancers. *Nat. Med.* 21 938–945. 10.1038/nm.3909 26193342 PMC4852857

[B30] Germain C. Gnjatic S. Tamzalit F. Knockaert S. Remark R. Goc J. (2014). Presence of B cells in tertiary lymphoid structures is associated with a protective immunity in patients with lung cancer. *Am. J. Respir. Crit. Care Med.* 189 832–844. 10.1164/rccm.201309-1611OC 24484236

[B31] Grum-Schwensen B. Klingelhofer J. Grigorian M. Almholt K. Nielsen B. S. Lukanidin E. (2010). Lung metastasis fails in MMTV-PyMT oncomice lacking S100A4 due to a T-cell deficiency in primary tumors. *Cancer Res.* 70 936–947. 10.1158/0008-5472.CAN-09-3220 20103644

[B32] Gunderson A. J. Coussens L. M. (2013). B cells and their mediators as targets for therapy in solid tumors. *Exp. Cell Res.* 319 1644–1649. 10.1016/j.yexcr.2013.03.005 23499742 PMC3743954

[B33] Guy T. V. Terry A. M. Bolton H. A. Hancock D. G. Shklovskaya E. Fazekas de St. Groth B. (2016). Pro- and anti-tumour effects of B cells and antibodies in cancer: a comparison of clinical studies and preclinical models. *Cancer Immunol. Immunother.* 65 885–896. 10.1007/s00262-016-1848-z 27222052 PMC11029718

[B34] Harper J. Sainson R. C. (2014). Regulation of the anti-tumour immune response by cancer-associated fibroblasts. *Semin. Cancer Biol.* 25 69–77. 10.1016/j.semcancer.2013.12.005 24406209

[B35] Heng T. S. Painter M. W. (2008). The immunological genome project: networks of gene expression in immune cells. *Nat. Immunol.* 9 1091–1094. 10.1038/ni1008-1091 18800157

[B36] Isella C. Terrasi A. Bellomo S. E. Petti C. Galatola G. Muratore A. (2015). Stromal contribution to the colorectal cancer transcriptome. *Nat. Genet.* 47 312–319. 10.1038/ng.3224 25706627

[B37] Jain R. K. (2013). Normalizing tumor microenvironment to treat cancer: bench to bedside to biomarkers. *J. Clin. Oncol.* 31 2205–2218. 10.1200/JCO.2012.46.3653 23669226 PMC3731977

[B38] Jia D. Liu Z. Deng N. Tan T. Z. Huang R. Y. Taylor-Harding B. (2016). A COL11A1-correlated pan-cancer gene signature of activated fibroblasts for the prioritization of therapeutic targets. *Cancer Lett.* 382 203–214. 10.1016/j.canlet.2016.09.001 27609069 PMC5077659

[B39] Kalluri R. (2016). The biology and function of fibroblasts in cancer. *Nat. Rev. Cancer* 16 582–598. 10.1038/nrc.2016.73 27550820

[B40] Kalluri R. Zeisberg M. (2006). Fibroblasts in cancer. *Nat. Rev. Cancer* 6 392–401. 10.1038/nrc1877 16572188

[B41] Karin N. (2010). The multiple faces of CXCL12 (SDF-1alpha) in the regulation of immunity during health and disease. *J. Leukoc. Biol.* 88 463–473. 10.1189/jlb.0909602 20501749

[B42] Kitamura T. Qian B. Z. Pollard J. W. (2015). Immune cell promotion of metastasis. *Nat. Rev. Immunol.* 15 73–86. 10.1038/nri3789 25614318 PMC4470277

[B43] Koning J. J. Mebius R. E. (2012). Interdependence of stromal and immune cells for lymph node function. *Trends Immunol.* 33 264–270. 10.1016/j.it.2011.10.006 22153930

[B44] Kraman M. Bambrough P. J. Arnold J. N. Roberts E. W. Magiera L. Jones J. O. (2010). Suppression of antitumor immunity by stromal cells expressing fibroblast activation protein-alpha. *Science* 330 827–830. 10.1126/science.1195300 21051638

[B45] Lenz G. Wright G. Dave S. S. Xiao W. Powell J. Zhao H. (2008). Stromal gene signatures in large-B-cell lymphomas. *N. Engl. J. Med.* 359 2313–2323. 10.1056/NEJMoa0802885 19038878 PMC9103713

[B46] Levental K. R. Yu H. Kass L. Lakins J. N. Egeblad M. Erler J. T. (2009). Matrix crosslinking forces tumor progression by enhancing integrin signaling. *Cell* 139 891–906. 10.1016/j.cell.2009.10.027 19931152 PMC2788004

[B47] Lossos I. S. Czerwinski D. K. Alizadeh A. A. Wechser M. A. Tibshirani R. Botstein D. (2004). Prediction of survival in diffuse large-B-cell lymphoma based on the expression of six genes. *N. Engl. J. Med.* 350 1828–1837. 10.1056/NEJMoa032520 15115829

[B48] Lu P. Weaver V. M. Werb Z. (2012). The extracellular matrix: a dynamic niche in cancer progression. *J. Cell Biol.* 196 395–406. 10.1083/jcb.201102147 22351925 PMC3283993

[B49] Mariathasan S. Turley S. J. Nickles D. Castiglioni A. Yuen K. Wang Y. (2018). TGFbeta attenuates tumour response to PD-L1 blockade by contributing to exclusion of T cells. *Nature* 554 544–548. 10.1038/nature25501 29443960 PMC6028240

[B50] Meyer P. N. Fu K. Greiner T. Smith L. Delabie J. Gascoyne R. (2011). The stromal cell marker SPARC predicts for survival in patients with diffuse large B-cell lymphoma treated with rituximab. *Am. J. Clin. Pathol.* 135 54–61. 10.1309/ajcpjx4bjv9nlqhy 21173124

[B51] Mionnet C. Mondor I. Jorquera A. Loosveld M. Maurizio J. Arcangeli M. L. (2013). Identification of a new stromal cell type involved in the regulation of inflamed B cell follicles. *PLoS Biol.* 11:e1001672. 10.1371/journal.pbio.1001672 24130458 PMC3794863

[B52] Moffitt R. A. Marayati R. Flate E. L. Volmar K. E. Loeza S. G. Hoadley K. A. (2015). Virtual microdissection identifies distinct tumor- and stroma-specific subtypes of pancreatic ductal adenocarcinoma. *Nat. Genet.* 47 1168–1178. 10.1038/ng.3398 26343385 PMC4912058

[B53] Mueller S. N. Germain R. N. (2009). Stromal cell contributions to the homeostasis and functionality of the immune system. *Nat. Rev. Immunol.* 9 618–629. 10.1038/nri2588 19644499 PMC2785037

[B54] Raz Y. Erez N. (2013). An inflammatory vicious cycle: fibroblasts and immune cell recruitment in cancer. *Exp. Cell Res.* 319 1596–1603. 10.1016/j.yexcr.2013.03.022 23567181

[B55] Reimann M. Lee S. Loddenkemper C. Dorr J. R. Tabor V. Aichele P. (2010). Tumor stroma-derived TGF-beta limits myc-driven lymphomagenesis via Suv39h1-dependent senescence. *Cancer Cell* 17 262–272. 10.1016/j.ccr.2009.12.043 20227040

[B56] Sanjabi S. Oh S. A. Li M. O. (2017). Regulation of the immune response by TGF-beta: from conception to autoimmunity and infection. *Cold Spring Harb. Perspect. Biol.* 9:a022236. 10.1101/cshperspect.a022236 28108486 PMC5453394

[B57] Sarvaria A. Madrigal J. A. Saudemont A. (2017). B cell regulation in cancer and anti-tumor immunity. *Cell Mol. Immunol.* 14 662–674. 10.1038/cmi.2017.35 28626234 PMC5549607

[B58] Shay T. Kang J. (2013). Immunological genome project and systems immunology. *Trends Immunol.* 34 602–609. 10.1016/j.it.2013.03.004 23631936 PMC4615706

[B59] Sheng J. Chen W. Zhu H. J. (2015). The immune suppressive function of transforming growth factor-beta (TGF-beta) in human diseases. *Growth Factors* 33 92–101. 10.3109/08977194.2015.1010645 25714613

[B60] Silzle T. Kreutz M. Dobler M. A. Brockhoff G. Knuechel R. Kunz-Schughart L. A. (2003). Tumor-associated fibroblasts recruit blood monocytes into tumor tissue. *Eur. J. Immunol.* 33 1311–1320. 10.1002/eji.200323057 12731056

[B61] Spano D. Zollo M. (2012). Tumor microenvironment: a main actor in the metastasis process. *Clin. Exp. Metastasis* 29 381–395. 10.1007/s10585-012-9457-5 22322279

[B62] Stelling A. Hashwah H. Bertram K. Manz M. G. Tzankov A. Muller A. (2018). The tumor suppressive TGF-beta/SMAD1/S1PR2 signaling axis is recurrently inactivated in diffuse large B-cell lymphoma. *Blood* 131 2235–2246. 10.1182/blood-2017-10-810630 29615404

[B63] Stover D. G. Bierie B. Moses H. L. (2007). A delicate balance: TGF-beta and the tumor microenvironment. *J. Cell. Biochem.* 101 851–861. 10.1002/jcb.21149 17486574

[B64] Tauriello D. V. F. Palomo-Ponce S. Stork D. Berenguer-Llergo A. Badia-Ramentol J. Iglesias M. (2018). TGFbeta drives immune evasion in genetically reconstituted colon cancer metastasis. *Nature* 554 538–543. 10.1038/nature25492 29443964

[B65] Tekin N. Omidvar N. Morris T. P. Conget P. Bruna F. Timar B. (2016). Protocol for qRT-PCR analysis from formalin fixed paraffin embedded tissue sections from diffuse large b-cell lymphoma: validation of the six-gene predictor score. *Oncotarget* 7 83319–83329. 10.18632/oncotarget.13066 27825111 PMC5347772

[B66] Turley S. J. Cremasco V. Astarita J. L. (2015). Immunological hallmarks of stromal cells in the tumour microenvironment. *Nat. Rev. Immunol.* 15 669–682. 10.1038/nri3902 26471778

[B67] Yeung T. L. Leung C. S. Wong K. K. Samimi G. Thompson M. S. Liu J. (2013). TGF-beta modulates ovarian cancer invasion by upregulating CAF-derived versican in the tumor microenvironment. *Cancer Res.* 73 5016–5028. 10.1158/0008-5472.can-13-0023 23824740 PMC3745588

